# Sediment textural characteristics and elemental distribution in the core sediments, Pullivasal and Kurusadai Island, Gulf of Mannar, Southeast coast of India

**DOI:** 10.1016/j.dib.2017.09.070

**Published:** 2017-10-02

**Authors:** P. Saravanan, S. Krishnakumar, P. Parthasarathy, Judith D. Silva, D. Pradhap

**Affiliations:** a Department of Geology, University of Madras, Guindy Campus, Chennai 600025, India; b Department of Energy, University of Madras, Guindy Campus, Chennai 600025, India

**Keywords:** Sediment texture, Coral islands, Gulf of Mannar, Core sediments, Elemental distribution

## Abstract

Two core samples were collected in order to assess the textual characteristics and elemental distribution of the sediments, from the lagoonal environment of Pullivasal and Kurusadai island, Gulf of Mannar, Southeast coast of India. The distribution of the organic matter and calcium carbonate is chiefly controlled by the coral debris, shell fragments and mangrove litters. The elemental distribution is controlled by natural process and other trace elements are controlled by anthropogenic land based activities.

**Specifications Table**

**Table:** 

Subject area	Sedimentology, Geochemistry
More specific subject area	Sediment geochemistry
Type of data	Table and Figure
How data was acquired	Grain size analysis, Total digestion and Atomic Absorption Spectrophotometer (Model no - ELICO SL 194)
Data format	Raw data, analyzed
Experimental factors	Sediment core samples were collected from coral islands using PVC pipes
Experimental features	Assess the concentration of elements using AAS and Grain size studies using an electronic sieve shaker
Data source location	Pullivasal and Kurusadai Islands of Gulf of Mannar, Tamil Nadu, India
Data accessibility	Data available within the article

**Value of the data**

• The depositional environmental condition and natural disaster events can be studied the rough sediment textural analysis.
• The relationship between elemental concentration, calcium carbonate (CaCO3) and organic matter (OM) content is helpful to identify the mode of elemental transport in the coral reef environment.
• The coral rubbles and lithoclastic sediments are explaining the intensity of eolian and marine process during the past.

## 1 Data

**Fig. 1:**
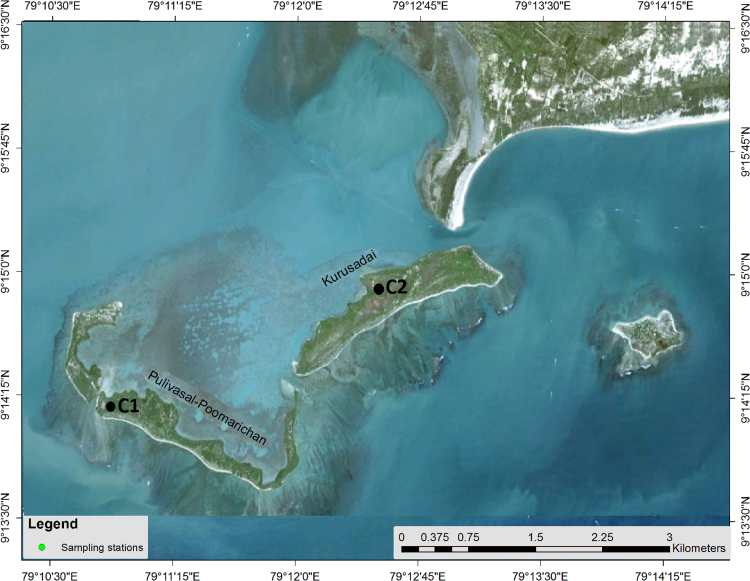
Study area map with core sample locations.

**Fig. 2:**
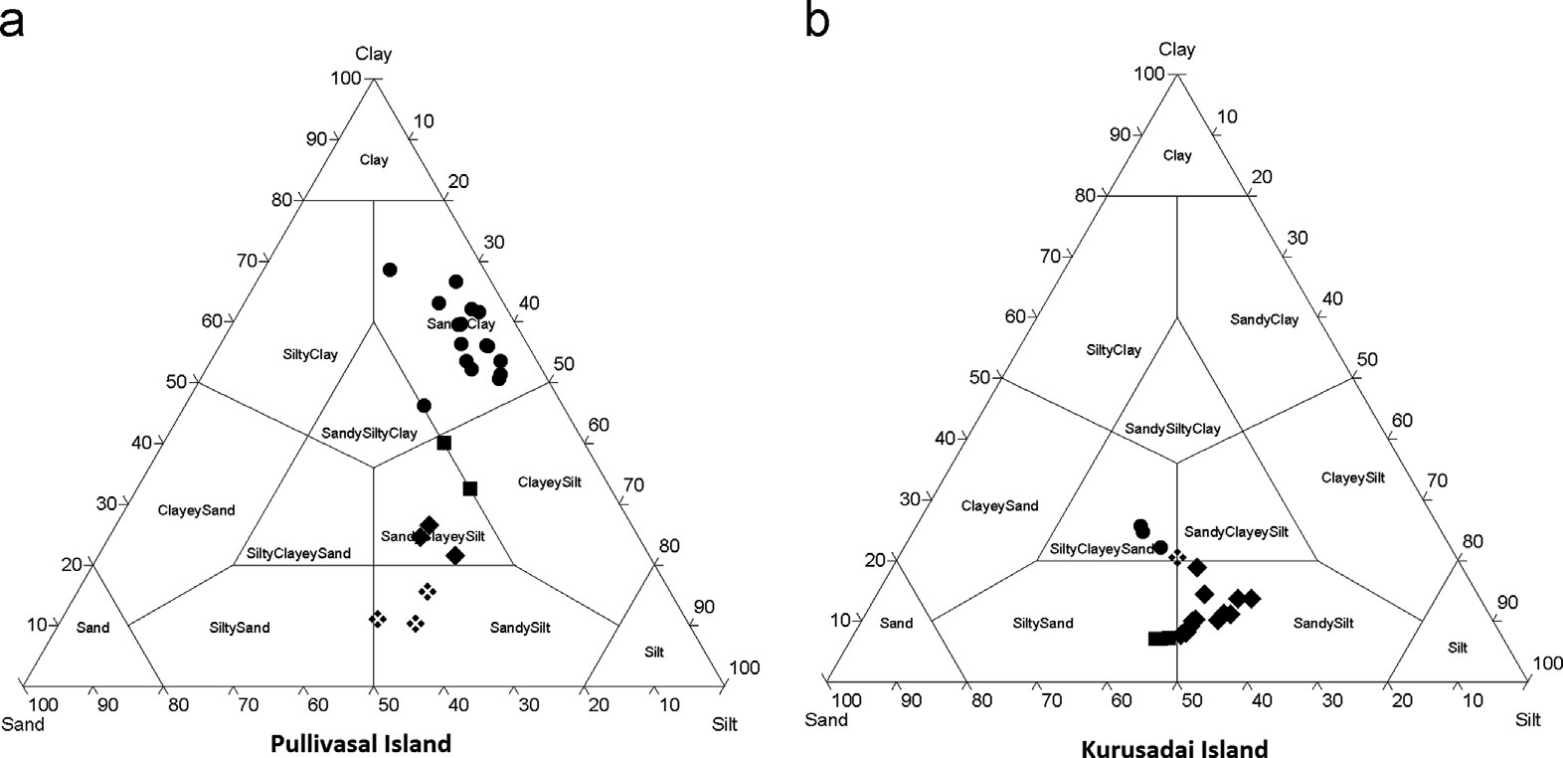
Ternary diagram for sand–silt–clay distribution.

**Fig. 3:**
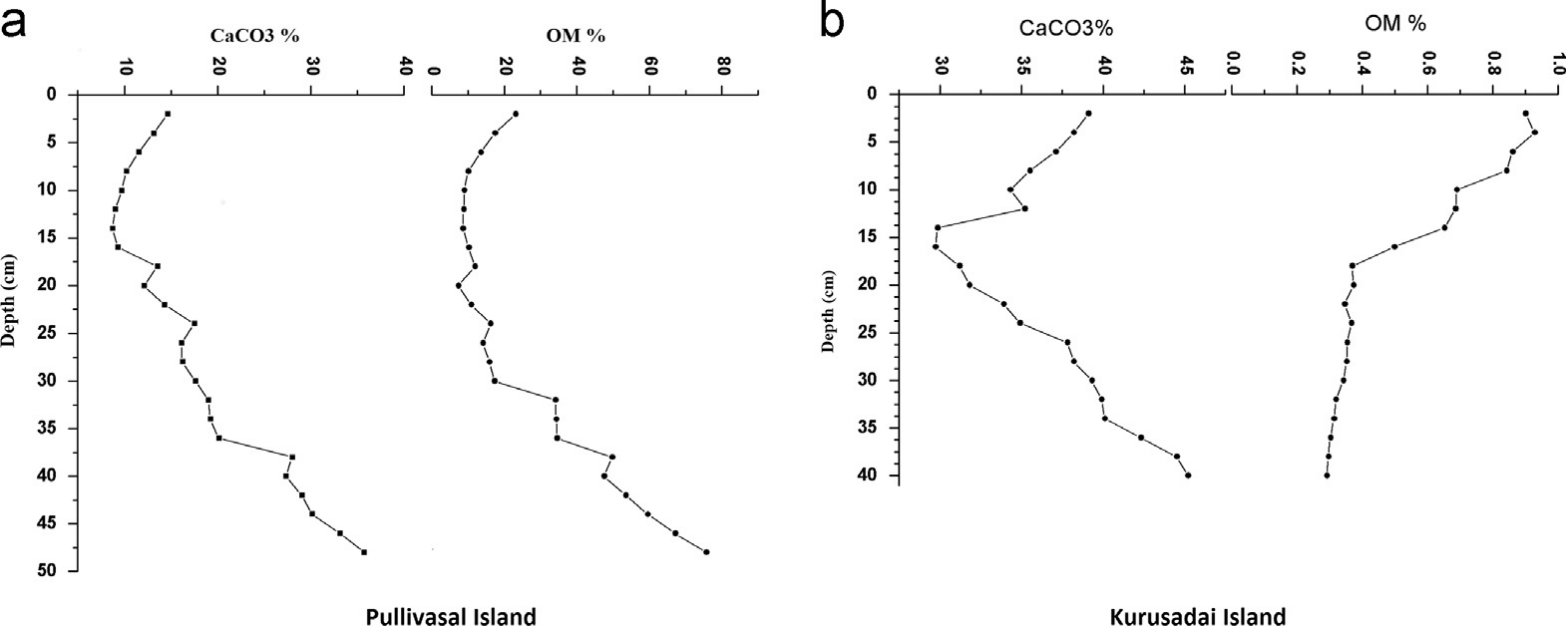
Vertical distribution of organic matter (OM) and calcium carbonate in the core sediments of the study area.

**Fig. 4:**
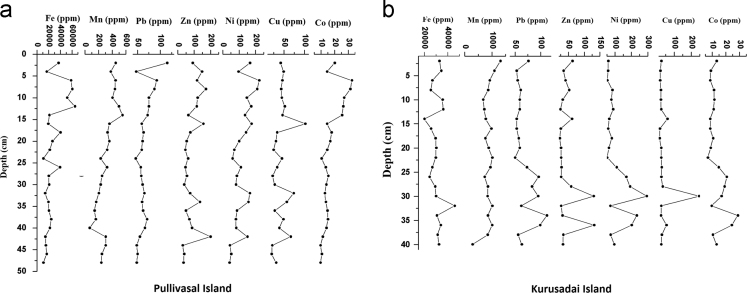
Vertical distribution of Fe, Mn and other trace elements in the core sediments of the study area.

The sampling location was chosen from Pullivasal and Kurusadai Islands of Gulf of Mannar (Fig. 1). Table 1, Table 2 is representing the sediment textural characteristics, CaCO_3_, Organic matter and elemental distribution of the core sediments of Pullivasal and Kurusadai Islands of Gulf of Mannar. The vertical distribution of the sediment textural characteristics, CaCO_3_, Organic matter and the elements were plotted in Fig. 2, Fig. 3, Fig. 4.

**Table 1:** Elemental concentration, textural charactrestics of sediment, Calcium carbonate (CaCO_3_) and Organic matter(OM) level of core sediments, Pullivasal Island, Gulf of Mannar, Southeast cost of India.

S.no	Subsample Interval	Fe	Mn	Pb	Zn	Ni	Cr	Cu	Co	Sand	Clay	Silt	CaCO_3_	OM
1	0–2	36,650	450	115	90.48	165	408	41.7	19.9	13.40	68.5	18.099	14.6	23.05
2	2–4	16,460	378	47	146.8	95.6	386.2	48.7	14.3	10.13	53.4	36.472	13.1	17.42
3	4–6	58,220	448	92	116.46	222	1077.8	45.7	32.5	7.82	59.5	32.678	11.5	13.45
4	6–8	60,340	438	87	170.91	209.6	1359.3	42.7	31.4	5.81	55.9	38.287	10.2	10.00
5	8–10	51,580	404	75	120.22	146.1	1269.4	44.4	26.8	5.19	53.4	41.408	9.7	8.93
6	10–12	64,790	498	75	115.05	173.1	960.1	51.8	26.2	5.09	62	32.908	9	8.76
7	12–14	21,140	550	72	64.06	134.8	900.5	46.2	25.3	4.98	66.5	28.52	8.7	8.57
8	14–16	19,290	358	59	155.07	175.1	981.3	101.6	14.3	5.89	56	38.108	9.3	10.13
9	16–18	40,070	330	63	75.92	141.1	1271.1	33	17.6	6.89	50.5	42.608	13.5	11.85
10	18–20	26,390	356	61	53.68	99.3	675.1	30	16.6	4.23	61.5	34.266	12.1	7.28
11	20–22	21,810	324	59	45.54	69.3	718.1	24.3	14.4	6.30	51.25	42.446	14.3	10.84
12	22–24	10,810	230	46	61.98	58.6	804.7	44.7	10.3	9.40	56.2	34.399	17.5	16.17
13	24–26	39,320	322	57	45.69	109.6	911.1	32.9	13.3	8.20	59.45	32.349	16.1	14.11
14	26–28	20,140	250	58	54.85	84.8	783.9	21.9	15.2	9.21	63	27.79	16.2	15.84
15	28–30	20,580	230	61	38.69	79.8	732.4	26.9	13.8	10.04	52.05	37.909	17.6	17.27
16	30–32	13,850	204	65	74.52	164.9	1115.9	73.6	12.7	19.80	46.05	34.149	19	34.06
17	32–34	19,420	164	60	134.37	155.7	1033.3	56.5	13.4	19.94	40.05	40.007	19.2	34.30
18	34–36	20,230	140	62	49.01	86	828.9	27.1	14.6	20.04	32.45	47.507	20.1	34.47
19	36–38	24,200	158	71	70.1	78.7	905	48.1	14.9	28.90	26.45	44.649	28	49.71
20	38–40	21,950	70	66	86.45	81.2	877.6	38.2	13.9	27.60	21.45	50.949	27.3	47.47
21	40–42	14,290	304	55	199.57	149.7	1445.3	66.4	11.2	31.10	24.45	44.449	29	53.49
22	42–44	15,060	304	48	29.41	42.3	837.6	21.2	9.5	34.60	15.45	49.948	30.1	59.52
23	44–46	16,430	248	50	38.14	50.6	849.3	19.5	10.5	39.01	10.2	50.789	33.1	67.10
24	46–48	10,970	238	48	34.26	40.3	809.6	30.5	9.5	44.01	10.9	45.089	35.7	75.70

Elemental concentration in ppm; Sand, Silt, Clay and Calcium carbonate level in percentage.

**Table 2:** Elemental concentration, textural charactrestics of sediment, Calcium carbonate (CaCO_3_) and Organic matter(OM) level of core sediments, Kurusadai Island, Gulf of Mannar, Southeast cost of India.

S.no	Subsample Interval	Fe	Mn	Pb	Zn	Ni	Cu	Co	Sand	Clay	Silt	CaCO_3_	OM
1	0–2	32,980	1308	76	62.47	58.2	23.4	13.2	42.663	24.55	32.787	39.1	0.5236
2	2–4	34,560	1084	53	34.12	56.7	18.2	8.9	42.448	25.6	31.952	38.2	0.5398
3	4–6	26,920	916	55	40.41	52.8	16.4	8.5	41.303	22	36.697	37.1	0.5003
4	6–8	25,640	856	61	52.1	83	20.8	11.2	39.809	20.36	39.831	35.5	0.4895
5	8–10	35,690	672	59	31.65	76.6	21.1	11.5	37.85	18.689	43.461	34.3	0.4008
6	10–12	36,200	712	59	24.22	87.6	23.1	11	38.98	14.321	46.699	35.2	0.3987
7	12–14	20,140	766	53	60.83	63.7	59.2	8.5	34.565	13.602	51.833	29.85	0.3789
8	14–16	25,830	970	53	25.29	57.5	14.9	8.5	32.589	13.61	53.801	29.72	0.2898
9	16–18	29,620	752	57	22.32	79.6	13.7	10.6	36.891	11.025	52.084	31.2	0.2145
10	18–20	30,260	872	59	25.97	61.4	22.7	8.8	37.801	11.123	51.076	31.8	0.2168
11	20–22	29,820	1008	50	26.64	51.6	22.8	6.8	39.201	10.025	50.774	33.9	0.2012
12	22–24	26,890	936	73	27.68	109.1	26.2	14.7	42.258	10.14	47.602	34.9	0.2132
13	24–26	24,580	726	96	26.61	167.8	23.5	20.6	42.856	9.898	47.246	37.8	0.2054
14	26–28	29,320	846	83	58.2	191.4	31.8	19	43.563	9.112	47.325	38.2	0.2047
15	28–30	30,110	834	95	129.93	293.2	244.3	16.8	44.598	8.089	47.313	39.3	0.1987
16	30–32	46,010	1006	62	26.47	70.9	23	9.5	44.698	8.011	47.291	39.9	0.1856
17	32–34	30,770	846	112	30.89	231.9	21.9	28.7	45.632	7.654	46.714	40.1	0.1821
18	34–36	34,170	1004	99	131.32	201.2	53.5	24.3	47.605	7.125	45.27	42.3	0.1758
19	36–38	31,390	838	56	33.61	72.3	26.6	10.4	48.806	7.023	44.171	44.5	0.1721
20	38–40	32,560	286	63	32.32	94.7	23	13.1	49.652	6.983	43.365	45.2	0.1692

Elemental concentration in ppm; Sand, Silt, Clay and Calcium carbonate level in percentage.

## 2 Experimental design, materials and methods

### 2.1 Sample collections

Two core samples were collected using PVC pipe and the retrieved core samples are transported to Department of Geology, University of Madras and keep the both cores at −5 °C. The total length of the cores is 50 and 40 cm respectively. The sub sample was separated at every 2 cm interval. The coarse grained coral rubbles were removed from a subsample manually.

### 2.2 Elemental analysis, textural characteristic studies, Determination of Organic matter and calcium carbonate (OM and CaCO_3_)

The textural characteristics of the sediments were clearly suggested the dominancy of fine fractions in the core sediments. This observation primary due to persistence of calm environment in the lagoon. The core sediments are dominated by sandy clay in Pullivasal Island and sandy silt in Kurusadai Island. Organic matter (OM) was determined by exothermic heating and oxidation with potassium dichromate and concentrated H_2_SO_4._ The excess amount of dichromate titrated with 0.5 N ferrous ammonium sulfate solution [3]. Calcium carbonate (CaCO3) and trace element analyses were performed as suggested by Loring and Rantala [2]. The maximum concentration of calcium carbonate in the lower part of the core may be due to the presence of small coral rubbles and coral sand. The same trend was also observed in organic matter content. The enrichment of organic matter in the down core region is chiefly derived from mangrove litters and decomposition sediment associated mangrove debris. 1 g of the sediment sample was placed in a Teflon bomb, 1 ml of aqua regia (AR grade HNO_3_: HCl; 1:3 v/v) was added, followed by 6 ml HF. The sealed bomb was submerged in boiling water bath (2 h and 30 min). After the bomb was removed from the water bath, the contents were added to 5.6 g of boric acid crystals in a 100 ml polypropylene standard flask. The flask was made up to volume (100 ml) with high purity distilled water (HPDW). The accuracy of the present analysis was checked with BCSS-1 analytical standard values and the recoveries of those elements were almost equal to that of the certified values. The laboratory results showed that the recovery efficiency ranges 92 to 97.5% of the studied elements. The limits of detection (LODs) of trace elements are 0.01 µg g^−1^ for Fe, Zn, Cr, Cu, Co, Ni, Cd, 0.02 µg g^−1^ for Mn and 0.05 µg g^−1^ for Pb. Fe and Mn concentrations in the core sediments are probably supplied through the riverine input and natural processes. The maximum concentration of Fe was noticed in few samples, and it may be due to the presence of Fe rich lithic fragments. The lead concentration may be due to coal incinerating power plants, commercial coal handling harbor activities in the southern part of the Gulf of Mannar and the application of leaded petrol around the coral ecosystem [1].
