# Supplementary material for: Sediment textural characteristics and elemental distribution in the core sediments, Pullivasal and Kurusadai Island, Gulf of Mannar, Southeast coast of India
Source: Data Brief. 2017 Oct 2;15:439–44. doi: 10.1016/j.dib.2017.09.070 (PMC5645517; doi:10.1016/j.dib.2017.09.070)
Supplement: Supplementary file 1 — Supplementary material [file mmc1.doc]

Date – 08/09/2017

From

Dr.S.Krishna Kumar

Institute for Ocean Management, Anna University

Chennai-25.

To

The Editor in Chief

Data in Brief

Dear sir/Madam

Sub- Conflict of interest for research Article – reg

The authors declare no conflict of interest connected with this manuscript entitled “**Sediment textural characteristics and Elemental distribution in the core sediments, Pullivasal and Kurusadai Island, Gulf of Mannar, Southeast coast of India”.**

Thanking you

Yours faithfully

Dr.S.Krishna Kumar
